# Endoscopic-assisted repair for sagittal synostosis

**DOI:** 10.3171/2021.1.FOCVID2044

**Published:** 2021-04-01

**Authors:** Matthew D. Smyth, Kamlesh B. Patel

**Affiliations:** 1 Department of Neurosurgery and; 2 Division of Plastic and Reconstructive Surgery, Washington University in St. Louis, Missouri

**Keywords:** craniosynostosis, endoscopy-assisted, strip craniectomy, sagittal

## Abstract

The craniofacial team at St. Louis Children's Hospital has been performing endoscopy-assisted synostosis surgery since 2006. Most infants with single-suture synostosis younger than 6 months of age are candidates. The sphinx position is used, with two incisions: one posterior to the bregma and one anterior to the lambda. The endoscope is incorporated primarily for epidural dissection and bone edge cauterization. Blood products are available but rarely needed with single suturectomies. Patients are managed on the floor after surgery and discharged to home on postoperative day 1, with helmet therapy coordinated and initiated immediately after surgery and continued until about 12 months of age.

The video can be found here: https://vimeo.com/513939623

## Transcript

This video highlights the endoscopic technique in a patient with sagittal synostosis.

**0:28 Patient Positioned for Surgery**. The patient is placed in the sphinx position on the Doro head holder, two IVs are placed, and also TXA is given preoperatively. The tube is secured, and emergency blood is available if needed.

**0:48 Surgical Procedure**. Two incisions are marked, one just posterior to the anterior fontanelle and one anterior to lambda, each 2.5 cm in length. Local anesthesia is infiltrated, and after adequate time for hemostasis the incisions are made sharply. Subgaleal dissection is performed in this bloodless plane, and this connects the two incisions.

At this point the periosteum is incised, subperiosteal dissection is performed all the way to the anterior fontanelle, and the appropriate burr hole is made by the neurosurgical service.

Curettes are then used to widen this osteotomy, and then a Kerrison punch to complete the osteotomy.

The osteotomy is complete for a total 2.5 cm, the same length as the incision. Gelfoam is placed for hemostasis. In a similar manner, a burr hole is made posteriorly and the osteotomy is widened.

**3:05 Endoscope Used to Visualize Dissection**. The 30° scope is inserted, angled up; the suction is placed in front of the scope to keep visibility and also to perform the dissection.

The epidural dissection is then completed all the way to the posterior incision. The posterior incision can be seen in this view.

**4:10 Removal of 2-cm-Wide Strip Involving Fused Sagittal Suture**. Tessier bone scissors are then used to perform the suturectomy. These scissors have a blunt tip to avoid injury.

The suturectomy is performed again about 2.5 cm in width. We originally performed a 5- to 6-cm suturectomy with barrel staves, but now have just gone to the single-strip craniectomy.

This segment of bone is removed, sometimes in one piece but often in two pieces.

**5:08 Hemostasis After Strip Craniectomy**. Hemostasis is now obtained over the dura, and then this J&B dural retractor is used to help cauterize the bone edges using the suction Bovie.

The J&B retractor really helps protect the scalp and the dura. The remainder of the suturetomy is performed under direct vision, all the way posteriorly to the lambdoid sutures and anteriorly to the anterior fontanelle.

**6:20 Closure and Postoperative Monitoring**. FloSeal is applied and the incisions are then closed, the galea and then skin. The CBC is checked 4 hours postoperatively, and the patient stays overnight on the neurosurgical ward service.

**6:29 References**^1–8^

## Author Contributions

Primary surgeon: both authors. Editing and drafting the video and abstract: both authors. Critically revising the work: Smyth. Reviewed submitted version of the work: both authors. Approved the final version of the work on behalf of both authors: Smyth.
